# Lower baseline autoantibody levels are associated with immune-related adverse events from immune checkpoint inhibition

**DOI:** 10.1136/jitc-2021-004008

**Published:** 2022-01-28

**Authors:** Nilasha Ghosh, Michael Postow, Chengsong Zhu, Deanna Jannat-Khah, Quan-Zhen Li, Greg Vitone, Karmela K Chan, Anne R Bass

**Affiliations:** 1 Hospital for Special Surgery/Weill Cornell Medical College, Department of Medicine, Division of Rheumatology, New York, NY, USA; 2 Melanoma & Immunotherapeutics Service, Department of Medicine, Memorial Sloan-Kettering Cancer Center, New York, NY, USA; 3 Department of Immunology, Microarray and Immune Phenotyping Core Facility, University of Texas Southwestern Medical Center, Dallas, TX, USA; 4 Hospital for Special Surgery, Department of Medicine, Division of Rheumatology, New York, New York, USA

**Keywords:** immunotherapy, immunity, humoral, autoimmunity, melanoma, antibody formation

## Abstract

**Introduction:**

Immune checkpoint inhibitors (ICI) are a novel cancer therapeutic that have been successful in treating advanced malignancies; however, they also cause immune-related adverse events (irAE). Given that some irAE are clinically similar to traditional autoimmune diseases, autoantibodies have been suggested as possible biomarkers of irAE. However, there are very little data on autoantibody investigation prior to ICI. Our aim was to determine if specific baseline autoantibodies were associated with irAE and see if changes in autoantibody concentration corresponded with irAE development.

**Methods:**

This study used data from an oncologic clinical trial of adaptive dosing combination ICI therapy in patients with advanced melanoma. Plasma was collected at baseline and 6 weeks after ICI initiation and tested in a microarray of 120 autoantigens commonly associated with autoimmune disease, as well as antinuclear antibody (ANA), rheumatoid factor (RF), and anti-cyclic citrullinated peptide antibody (anti-CCP). Autoantibody concentrations were compared between patients experiencing an organ-specific event versus not. Heatmaps, volcano plots and hierarchical clustering were used to determine autoantibody concentration differences among irAE patient clusters as defined by signal intensity of autoantibodies. Kaplan-Meier curves were created and a log-rank test was performed to assess differences in survival.

**Results:**

The microarray analysis demonstrated that patients who experienced specific irAE had fewer differentially expressed autoantibodies at baseline than those that did not have those specific irAE, and a greater fold change (FC) in antibody concentration from baseline to 6 weeks corresponded with specific irAE development. However, no autoantibodies were identified as being predictive of specific events. Time to first irAE was less than 6 weeks in 69% of patients, and these patients had less autoantibodies at baseline. Considering ANA, RF and CCP autoantibodies, there were no significant differences between the seropositive and seronegative patients in irAE development, severity, timing or survival.

**Conclusion:**

Patients with low autoantibody concentrations at baseline as well as a greater FC in autoantibody concentration over 6 weeks developed more distinct organ-specific irAE. This may suggest differences in the balance of cellular immunity and humoral pathways that are relevant in the pathogenesis of irAE, though further investigation is needed.

## Introduction

Immune checkpoint inhibitors (ICI), such as anti-cytotoxic T-lymphocyte-associated protein-4 (anti-CTLA-4), anti-programmed cell death protein-1 (anti-PD-1) and anti-programmed death ligand-1 (anti-PD-L1), have revolutionized the treatment of advanced malignancies, often succeeding when chemotherapies have failed. They have been approved for the treatment of various malignancies, such as metastatic melanoma, non-small cell lung cancer (NSCLC) and urothelial cancers, as well as for adjuvant treatment for some surgically resected high-risk cancers. They primarily function by blocking T-cell inhibition, thus boosting the antitumor T-cell immune response.^1^ T cells are particularly effective agents of antitumor immunity due to their capacity to directly recognize and kill antigen-expressing cells and their ability to orchestrate diverse immune responses that integrate adaptive and effector mechanisms.^1^ PD-1 ligands are upregulated in many cancers, and PD-1 is highly expressed on tumor-infiltrating lymphocytes; thus, inhibiting this pathway has been particularly fruitful for an enhanced T-cell antitumor response.^2^ However, this ongoing activation of T cells also causes off-target autoimmune side effects, termed immune-related adverse events (irAE), which can affect almost any organ system. Single-cell studies have shown the proliferation and accumulation of T cells, particularly CD8+ T cells, within organs affected by irAE, such as in colitis^3^ and arthritis.^4^ IrAE occur in as many as 80% of patients, and are especially frequent in patients treated with combination anti-CTLA-4/PD-1.^5 6^ While some irAE are subclinical or mild, others can be life-threatening,^7^ and it is not clear which patients are most at-risk for significant toxicity. To date, only a few biomarkers of irAE have been identified,^8^ but their ability to predict who will have toxicity is still in question. Given that some irAE are clinically similar to traditional autoimmune diseases, such as rheumatoid arthritis (RA) or Hashimoto’s thyroiditis, organ-specific autoantibodies have been suggested as possible biomarkers of irAE development.

Autoantibodies, which are products of the humoral immune system driven by B cells, have been implicated in some traditional autoimmune diseases as being pathogenic and causing inflammation.^9^ However, autoantibodies in isolation are not sufficient to cause autoimmune disease and, in many conditions, act as biomarkers rather than effectors of disease. Disease-specific autoantibodies often precede clinical disease by many years, particularly in the case of RA and systemic lupus erythematous (SLE).^10 11^ The frequency of disease-specific autoantibodies in the general population, other than antinuclear antibody (ANA), is quite low. In patients being treated with combination ICI therapy, an early increase in plasmablasts, the precursors to plasma cells that release antibodies, has been demonstrated in patients who go on to develop severe grade irAE.^12^ Certain antibodies have also been found in patients with irAE, particularly among some dermatologic, endocrine and neuromuscular irAE.^13–16^ One retrospective study by Toi *et al* found that in a cohort of patients with NSCLC treated with PD-1/L1 inhibition, there was an association of baseline autoantibodies, particularly ANA and rheumatoid factor (RF), with irAE development and improved progression-free survival.^13^ Furthermore, pre-existing thyroid autoantibodies, such as anti-thyroid peroxidase (anti-TPO) or antithyroglobulin, were associated with thyroid dysfunction. However, other studies have yielded conflicting results,^17–19^ which may be due to lack of standardization in autoantibody testing, retrospective study design, or the heterogeneity of patient populations. While it appears that irAE seem to be a result of enhanced T-cell activation, the humoral immune system may play a supporting role. To investigate if autoantibodies could serve as predictive biomarkers, a homogenous cohort of patients should be tested for autoantibodies prior to receiving ICI, and then followed clinically to observe the occurrence of irAE. By using plasma samples that were collected from patients enrolled in a clinical trial of adaptive dosing combination ICI for advanced melanoma (the Adaptively Dosed ImmunoTherapy Trial, ADAPT-IT),^20^ we aimed to establish an association of baseline connective tissue disease-associated autoantibodies and the development of organ-specific irAE.

## Methods

### Patient and data collection

This study included 60 patients with unresectable stage III or IV melanoma enrolled in an investigator-initiated phase II clinical trial of combination ICI therapy for the treatment of melanoma (NCT03122522), in which patients received two doses of ipilimumab (anti-CTLA-4) 3 mg/kg+nivolumab (anti-PD-1) 1 mg/kg every 3 weeks, followed by nivolumab alone at 480 mg every 4 weeks if they had evidence of favorable treatment benefit measured as decreased tumor burden by RECIST V.1.1 criteria.^21^ Maintenance nivolumab was continued until unacceptable toxicity or confirmed disease progression. Disease progression was defined by worsening of a tumor’s size or number of metastases. Patients were excluded if they had received ICI therapy for unresectable stage III/IV melanoma prior to this trial, and patients stopped treatment due to excessive toxicity or withdrawal of consent. Clinical information was collected on all patients and included demographics, number of ICI cycles received, progression-free survival, overall survival and all information regarding adverse events (AE), including timing, severity and likelihood that the AE is attributable to the ICI (definite, probable, possible, unlikely, unrelated). The median follow-up was 25 months. AE occurring during the trial were categorized according to organ system involvement, and severity was graded on a scale of 1–5 according to Common Terminology Criteria for Adverse Events V.4.0 guidelines, with grade 5 corresponding to death.^22^ For this study, grade 3–5 AEs are designated as ‘severe’. The irAE of interest in this study were: hepatic, gastrointestinal (eg, diarrhea and colitis), dermatologic, arthralgia/arthritis (joint inflammation), myocarditis, myositis/myalgia (muscle inflammation), sicca (glandular dysfunction of the eyes and salivary glands causing excessive dryness) thyroid and non-thyroid endocrinopathy (encompassing pituitary failure, diabetes mellitus and adrenalitis). Other AEs were not included in this study due to rare occurrences or low attribution as ICI toxicity. Only irAE that were determined to be definitely and/or probably related to the ICI treatment in this clinical trial were included for our analysis. Our study used deidentified clinical data and plasma collected in the context of this prospective clinical trial.

### Plasma sample analysis

Samples were collected at baseline prior to ICI treatment and at week 6 after treatment initiation. Blood was collected from patients in BD sodium heparin tubes, which contained a Ficoll gel layer. After centrifugation, the plasma supernatant was saved and stored in tubes at −80°C awaiting further analysis. Specimens were later thawed and aliquoted for the analyses described below.

#### Autoantigen microarray

Plasma was sent to the University of Texas Southwestern Medical Center core laboratory for an autoantigen microarray, a fluorescent-based multiplex assay for the detection of 120 autoantibodies, both IgG and IgM (online supplemental table 1). This array is not only enriched for autoantigens important in connective tissue diseases such as SLE, but also includes several antigens associated with irAE in the prior literature, such as anti-TPO in thyroid disease, antiglutamic acid decarboxylase in diabetes, perinuclear antineutrophil cytoplasmic antibody in colitis and vasculitis, as well as inclusion of various cytokines and other antigens implicated in autoimmune conditions such as Sjogren’s disease, autoimmune hepatitis and inflammatory myositis.

10.1136/jitc-2021-004008.supp1 Supplementary data

Plasma samples were pretreated with DNAse-I and diluted for autoantibody profiling. The diluted serum samples were incubated with the autoantigen arrays, and the autoantibodies that bound were extracted with conjugated anti-mouse IgG and IgM to provide immunofluorescence signal intensities. Slides were scanned using a GenePix 4200A instrument (molecular devices) and GenePix Pro (V.7, molecular devices) software was used to measure the signal intensities for IgG and IgM to produce net fluorescence intensities (NFI) and signal-to-noise ratios (SNR) for each antigen. SNR≥3 were considered true signals from background noise and antibodies with SNR<3 in more than 90% of all samples were filtered out. An antibody score (ABS), a quantitative measurement of the binding capacity of each antibody with the corresponding autoantigen, was generated for each antibody using the following formula: ABS = log_2_[(NFI×SNR)+1], and the data were normalized by robust linear model approach using internal positive controls.

#### Immunofluorescence and ELISA testing

Plasma was tested for ANA via immunofluorescence, reported as negative or positive, as well as RF (RF>14 IU/mL is positive) and anti-cyclic citrullinated peptide antibody (anti-CCP, CCP>20 U/mL is positive) via ELISA.

#### Immunoglobulin measurement

Baseline immunoglobulin levels (IgG and IgM) were measured for each patient using quantitative nephelometry.

### Statistical analyses

#### Autoantigen microarray

Comparisons of categorical variables were completed using Fisher’s exact tests, whereas continuous variables were compared using Mann-Whitney U test or Kruskal Wallis test for multiple groups. Two-tailed non-parametric tests were used to compare ranks or differences between groups. Differential expression of IgG and IgM, noted as fold changes (FCs) between groups with adjusted p<0.05, was measured between those that experienced an irAE versus those that did not experience the irAE, irAE occurrence before or after 6 weeks and irAE severity. Antigens with noted differential expression were then isolated and analyzed to assess for any relationship to prespecified clinical irAE. Heatmaps, volcano plots and violin plots were generated by Pheatmap and plotting packages in R (V.4.05). Volcano plots illustrated the differential expression of IgG and IgM in the autoantigen microarray based on irAE versus non-irAE, with a p value set to 0.05 (horizontal dotted line in plots). Data were demonstrated as dots representing individual antigens. Those showing negative log_2_ (FC) indicate higher antibody levels in the non-irAE group and dots showing positive log_2_ (FC) indicate higher antibody levels in the irAE group. Colored dots signified antigens with differential expression meeting significance. Violin plots were then created to identify the specific antigens with significant differential signal expression for each organ-specific irAE and for timing of irAE. Heatmaps of antigens with differential expression were then paired with clinical data to identify associations of signal intensity with irAE. For a secondary analysis, samples were classified into four subgroups (low, slightly low, moderate, high) using differentially expressed antigens by hierarchical clustering algorithm in hclust R package. After identification of those autoantibody-based subgroups, occurrence of irAE among these clusters were evaluated for each group to identify antibody levels that may be at risk for irAE.

#### Immunofluorescence and ELISA

Patients were categorized into ‘antibody positive’ or ‘antibody negative’ groups based on the presence or absence of ANA, RF and/or anti-CCP at baseline. Comparisons between groups were performed using Fisher’s exact test, Χ^2^ tests for categorical variables and Wilcoxon rank-sum or two sample t-test for continuous variables. Kaplan-Meier curves were used to analyze time to irAE, both for first irAE and for only severe irAE, stratified by seropositivity at baseline. Progression-free survival, defined as time to disease progression and/or death, and overall survival, defined as time to death from any cause, were stratified by seropositivity at baseline and analyzed with Kaplan-Meier curves and log-rank testing. Progression-free survival and overall survival analyses only included patients with stage IV melanoma. All analyses were performed in STATA V.16.1 (College Station, Texas, USA), and all p values were two sided with statistical significance evaluated at the 0.05 alpha level.

#### Immunoglobulin measurement

Patients were then divided into quartiles based on their total IgG and IgM levels and two-tailed Fisher’s exact was used to analyze differences in clinical characteristics.

## Results

### Patient characteristics and AEs

Patient demographics and characteristics are displayed in table 1. Of the 60 patients included in the study, the median age was 63 (IQR 51, 70) with the majority of patients being male (63%) and having stage IV melanoma (85%). Patients received a median of 5.5 total ICI cycles (IQR 3, 18.5) and experienced a median of 3 (IQR 2, 5) irAE of interest. All patients experienced irAE; however, 5 patients (8.3%) did not experience any definite or probable irAE of interest, 27 (45%) only experienced grade 1–2 (mild/moderate) irAE and 28 (46.7%) experienced a grade 3–5 (severe) irAE. Two of the three patients who had developed myocarditis died (grade 5) of their irAE.

**Table 1:** Patient characteristics

Age, median (IQR) (SD)	63 (51, 70)
Sex, male n (%)	38 (63)
Cancer stage, n (%)	
Stage III	9 (15)
Stage IV	51 (85)
Melanoma type, n (%)	
Cutaneous	37 (61.7)
Uveal	1 (1.7)
Acral	1 (1.7)
Mucosal	16 (26.7)
Unknown	5 (8.3)
Cycles of ICI received, median (IQR)	5.5 (3, 18.5)
Total number of irAE, median (IQR)	3(2, 5)
Max irAE grading, n (%)	
None	5 (8.3)
Mild (grade 1–2)	27 (45)
Severe (grade 3–5)	28 (46.7)
Timing to first irAE, n (%)	
<6 weeks	38 (63)
≥6 weeks	17 (28)
Time to first severe irAE, n (%)	
<6 weeks	6 (21)
≥6 weeks	22 (79)
ANA positivity, n (%)	
Baseline	14 (23.3)
6 weeks	14 (23.3)
Any timepoint	19 (31.7)
RF positivity, n (%)	
Baseline	4 (6.7)
6 weeks	4 (6.7)
Any timepoint	6 (10)
Anti-CCP positivity, n (%)	
Baseline	0
6 weeks	1 (2)
Any timepoint	1 (2)
RF, CCP or ANA positive at baseline, n (%)	17 (28)
RF, CCP or ANA positive at any timepoint, n (%)	24 (40)

ANA, antinuclear antibody; anti-CCP, anti-cyclic citrullinated peptide; ICI, immune checkpoint inhibitor; irAE, immune-related adverse event; RF, rheumatoid factor.

**Figure 1:**
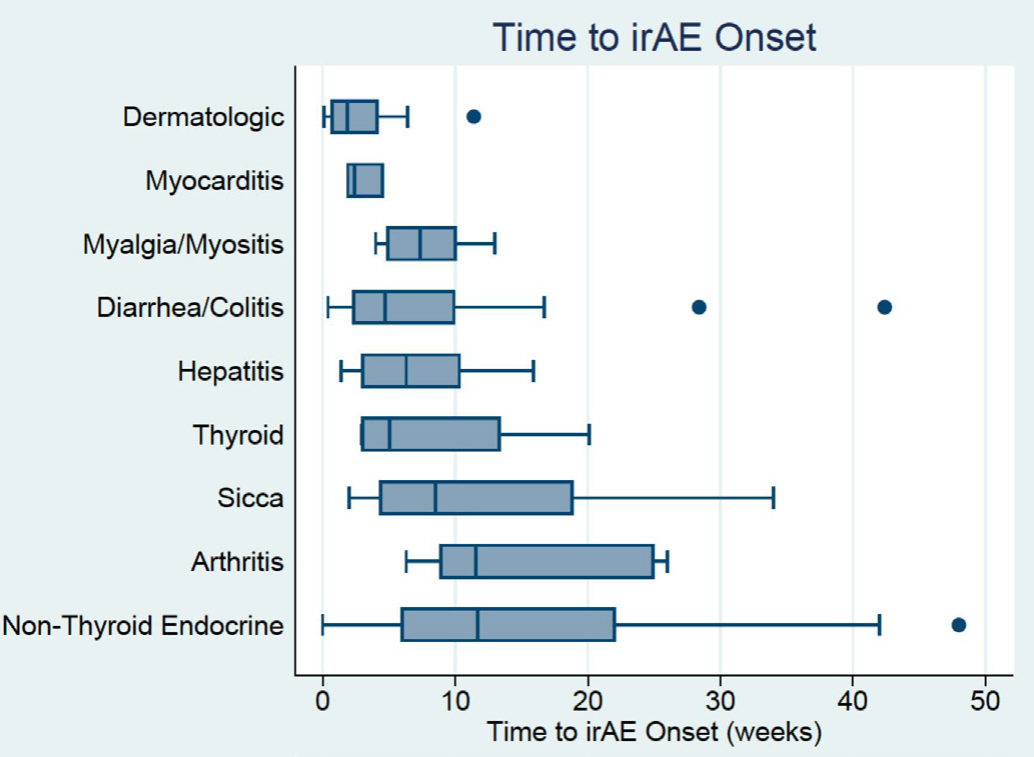
Boxplot of time to onset (weeks) of immune-related adverse events (irAE) based on organ system involved. Patients could have experienced multiple irAE either concurrently or sequentially.

Most patients (63%) experienced an irAE within the first 6 weeks of treatment (figure 1), although among the patients with severe irAE, the majority (79%) experienced them after 6 weeks. Patients often experienced multiple irAE, either concurrently or sequentially. Organ systems involved early included dermatologic (median 2.4 weeks), myocarditis (median 2.4 weeks), hepatic (mean 5.3 weeks) and diarrhea/colitis (6.3 weeks). Later events were noted to be arthritis/arthralgia (median 11.5 weeks) and non-thyroid endocrinopathies (median 11.7 weeks); however, the range of timing for irAE was variable as demonstrated by the boxplot.

### Autoantigen microarray

**Figure 2:**
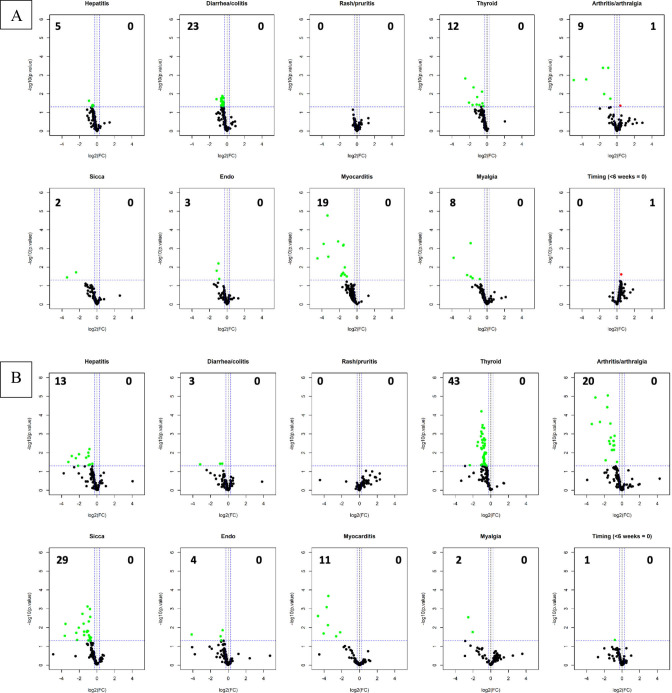
Volcano plots displaying baseline IgG (A) and IgM (B) antibodies in the autoantigen microarray based on immune-related adverse events (irAE) versus no irAE. Data showing negative log_2_(fold change (FC)), or left side of plot, indicate higher signal intensities in the no irAE group and data showing positive log_2_(FC), or right side of plot, indicate higher signal intensities in the irAE group with the numbers in the top corners numerating the number of significant antibodies that are differentially expressed with a p value of 0.05 (horizontal dotted line). Last panel displays timing of first irAE (earlier events with less signal intensity).

Of the 60 patients in the clinical trial, all had baseline plasma and 51/60 (85%) had plasma drawn at 6 weeks. Figure 2A, B displays differential expression of baseline IgG and IgM antibodies in relation to organ-specific irAE and to irAE timing (<6 or >6 weeks after ICI initiation). Patients who experienced irAE had lower levels of differentially expressed baseline IgG autoantibodies compared with those not experiencing that specific irAE. There was a trend toward more differentially expressed baseline autoantibodies in patients who experienced irAE later (>6 weeks) than earlier (<6 weeks). IgM volcano plot results showed similar patterns.

Violin plots displaying the differentially expressed autoantibodies in relation to organ-specific irAE are shown in online supplemental figure 1A–J. We did not find a significant association between organ-specific irAE and autoantibodies traditionally associated with autoimmune diseases in those targeted organs (eg, thyroid antibodies with thyroid irAE).

**Figure 3:**
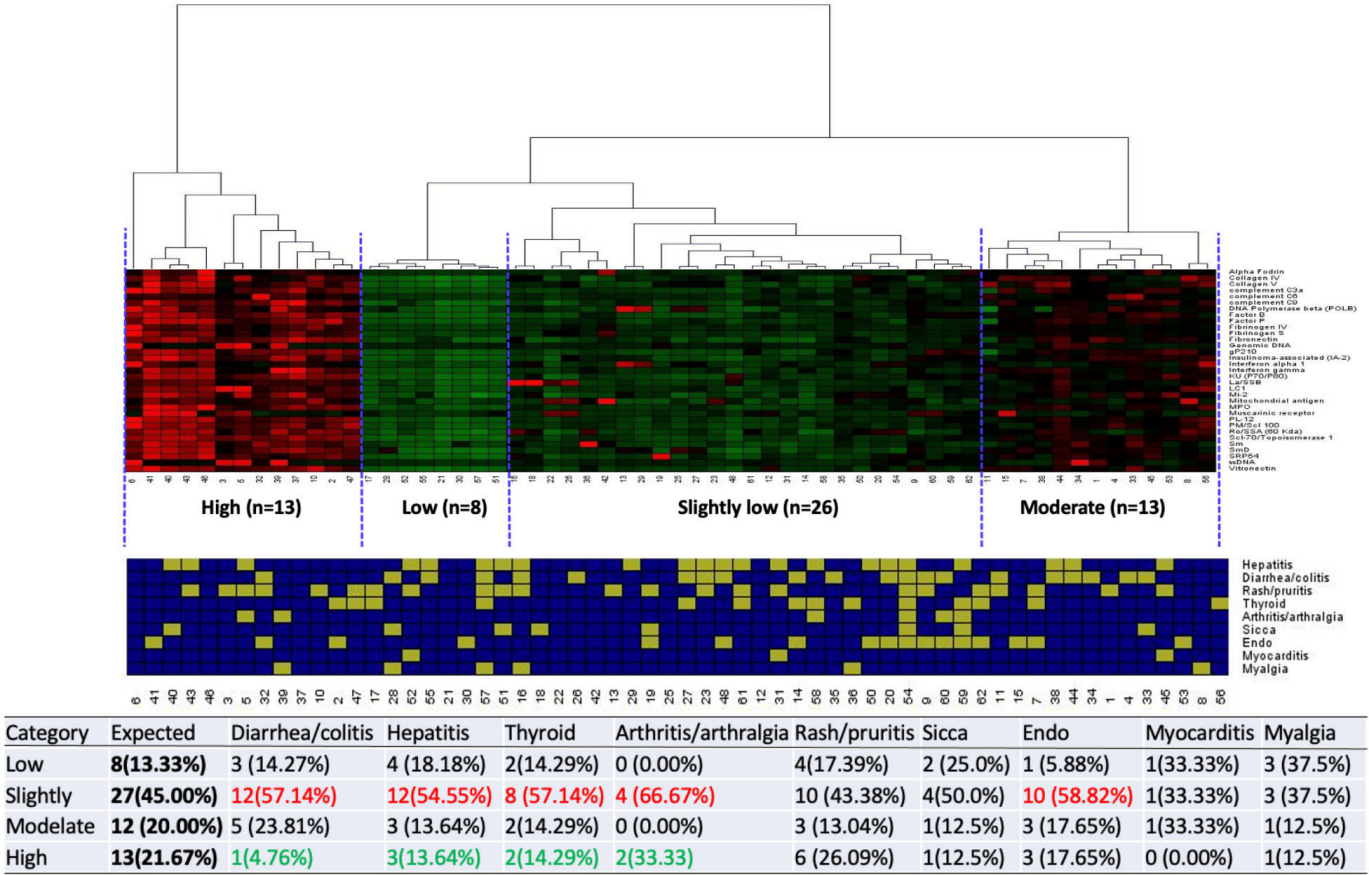
Hierarchical cluster and k means analysis of patients based on concentrations of differentially expressed baseline IgG antibodies for organ-specific events. Highlighted percentages indicate statistically significant differences within clusters (red=more common than expected, green=less common than expected).

Using hierarchical clustering, patients were divided into four clusters based on their overall baseline IgG signal intensity for differentially expressed autoantibodies—high signal intensity (13 patients), moderate signal intensity (12 patients), slightly low signal intensity (27 patients) and low signal intensity (8 patients) (figure 3). Significant differences in the irAE experienced among the clusters are highlighted. Patients with high signal intensity were less likely to experience diarrhea/colitis, hepatitis, thyroiditis and arthritis/arthralgia.

**Figure 4:**
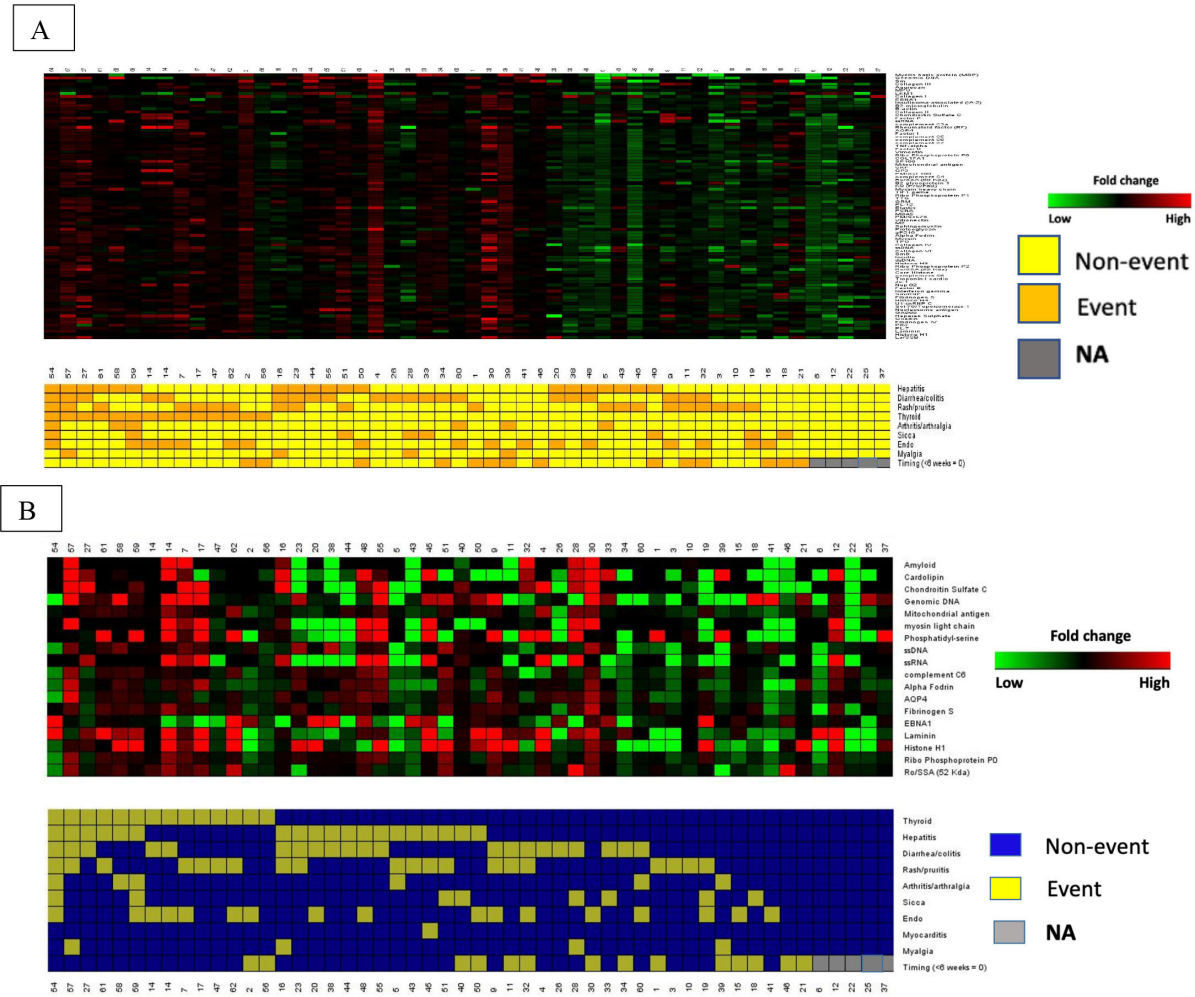
(A, B) Supervised clustering of patient immune-related adverse events (irAE) (event versus non-event) and fold changes in differentially expressed IgM (A) and IgG (B) antibodies from baseline to 6 weeks (green=negative fold changes, red=positive fold changes). Patients with no irAE (NA=gray) on the right with the lowest fold changes over time.

Changes in IgG and IgM concentration from baseline to 6 weeks of differentially expressed antibodies among are shown in figure 4A, B. Higher FCs for both IgG and IgM were associated with a greater number of distinct irAE.

### Immunofluorescence and ELISA testing

Results of ANA, RF and CCP testing are shown in table 1. Only 17 patients (28%) had any of these antibodies present at baseline, 1 of whom had both ANA and RF; 24 patients (40%) were noted be seropositive at any timepoint (baseline and/or 6 weeks). Seven patients seroconverted to positive and five patients seroconverted to negative, with nineteen seropositive at week 6. Patients who were ‘ANA/RF/CCP negative’ at baseline were similar in age, sex, number of irAE, irAE severity and irAE timing as those who were ‘ANA/RF/CCP positive’ at baseline (online supplemental table 2A). ‘ANA/RF/CCP-negative’ patients experienced more thyroid irAE compared with ‘ANA/RF/CCP-positive’ patients, with none of the seropositive patients experiencing a thyroid irAE (p=0.006, online supplemental table 2B).

**Figure 5:**
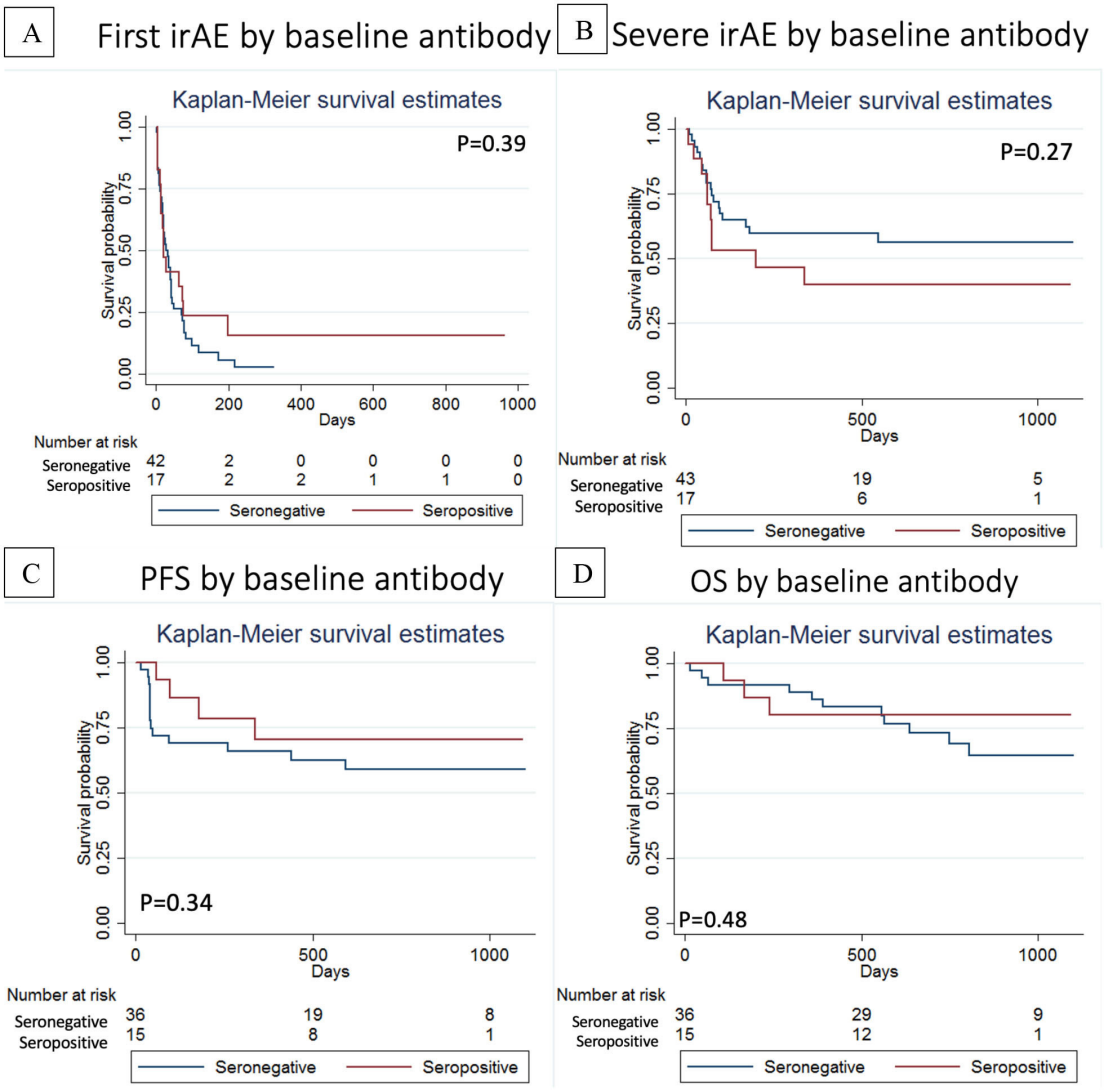
(A) Time to first immune-related adverse event and (B) Time to first severe immune-related adverse event stratified by baseline seropositivity (ANA, RF and/or CCP). (C) Progression-free survival and (D) overall survival (OS) stratified by baseline seropositivity (ANA, RF and/or CCP).

There was no association between baseline seropositivity for ANA/RF/CCP and time to first irAE (figure 5A) or time to first severe irAE (figure 5B). In fact, 3/17 (17.6%) of the patients who were ANA/RF/CCP positive at baseline experienced no irAE (vs 2/43 or 4.6% of seronegative patients). There was no statistically significant difference in progression-free survival or overall survival for the patients with stage IV cancers who were ‘ANA/RF/CCP positive’ versus ‘ANA/RF/CCP negative’ at baseline (figure 5C, D).

### Immunoglobulin measurement

Women had higher IgG levels at baseline (p=0.03). Differences in baseline levels of total IgG and total IgM were not associated with irAE timing, cancer histology, stage or max irAE severity. There were no differences in progression-free survival or overall survival between the highest quartiles and the lowest quartiles (online supplemental figure 2). Normalization of the results of the autoantigen microarray by baseline total IgG and IgM did not significantly alter our results.

## Discussion

Our microarray data demonstrated lower levels of differentially expressed autoantibodies at baseline in patients who developed organ specific irAE versus those who did not, while there was a greater FC in differentially expressed autoantibodies between the baseline and 6-week timepoint in these patients. We also found that patients with fewer autoantibodies had an earlier onset (<6 weeks) of their irAE. Adjusting for baseline total IgG/IgM levels did not qualitatively change our results, suggesting that our findings relate to the specific antigens tested, rather than overall levels of antibody production. These findings could suggest that patients with baseline autoantibodies have tolerance mechanisms in place that ‘protect’ them from ICI toxicity. It is well known that the immune system produces natural autoantibodies that recognize a variety of self-antigens that play a role in homeostatic antigen clearance. However, in certain immune environments, specific autoantibodies may present at higher levels and be linked to pathogenic conditions, such as in RA, Sjogren’s syndrome or SLE.^23^ Our 120-autoantigen microarray was, by design, heavily weighted toward autoantibodies associated with these rheumatic diseases. It is possible that said immune environments and/or genetic factors associated with prototypical autoantibody production (eg, certain HLA-DR alleles) are underrepresented among patients who experience irAE. It also may support the idea that irAE are driven by a different mechanism than traditional rheumatic diseases in which autoantibodies can induce inflammation through the formation of immune complexes, macrophage activation and complement fixation.^9^

Pairing the microarray data of FCs in autoantibody concentration over the 6 weeks with the clinical data, we found that patients with organ-specific irAE had higher FCs in autoantibody levels from baseline to 6 weeks. This reinforces the baseline autoantibody data where patients with low autoantibodies at baseline and the highest FC over time experienced more events. This may suggest these humoral responses in irAE are reactive, or correlative, rather than causative. This finding was also suggested by Das *et al* with a study that found that combination treatment-induced changes in B cells preceded and correlated with frequency and timing of irAE, specifically a decline in circulating B cells and an increase in CD21^10^ B cells and plasmablasts.^12^ A separate study investigating the humoral immune response to vaccinations in murine models treated with anti-PD1 suggested an altered T follicular helper cell–B-cell interaction that resulted in increased B activation capacity.^24^

We found no association between baseline autoantibodies and irAE severity, which differs from the results of Gowen *et al*.^25^ Those investigators used a human proteome array encompassing 19,000 full-length proteins to identify baseline autoantibodies in patients with melanoma initiating ICI. Using support vector machine classification model, they were able to identify a baseline autoantibody profile (incorporating discriminatory autoantibodies at both higher and lower levels) that distinguished patients who went on to develop high grade irAE (although these were not the prototypical autoantibodies associated with autoimmune disease). Their study did not attempt to identify autoantibody biomarkers of organ-specific irAE.

Baseline ANA, RF and CCP were not associated with organ specific irAE development, timing or severity. In fact, seropositive patients were less, rather than more, likely to have thyroid irAE than seronegative patients (p=0.006). Several other studies have also failed to find an association between commercially tested autoantibodies and irAE.^17 18 26^ In contrast, Toi *et al* demonstrated that, among patients with NSCLC treated with anti-PD-1 therapy, having a positive ANA, RF or thyroid autoantibody at baseline was strongly associated with irAE development compared with being seronegative (73% vs 45%, p=0.002).^13^ This finding was particularly driven by patients with a positive RF (39% vs 17%, p=0.006). This suggests that the relationship between autoantibodies and irAE may vary depending on the primary cancer (NSCLC vs melanoma), ICI regimen (monotherapy vs combination), population genetics and/or environmental factors such as smoking. Our study, which prospectively ascertained toxicity, is unique compared with previous studies done by retrospective toxicity attribution, which may introduce bias and enrich for some irAE. This is especially prevalent in some retrospective studies that are enriched in thyroid irAE^13 16 27^ or arthritis irAE^28 29^ that report a high prevalence of autoantibodies.

It should be noted that our patients did not have any autoimmune diseases at baseline, and that patients with known autoimmune diseases will likely respond differently to ICI therapy than the patients included in this study. Previous studies have demonstrated that up to one-half of patients with pre-existing autoimmune disease experience a flare of their underlying disease and one-third go on to develop a new irAE,^30 31^ though the risk of flare is likely different for different autoimmune diseases. It is also unclear what happens serologically over time as large population studies are lacking.

Our study has several strengths. This is the largest single-tumor-type series, where clinical data and patient autoantibody specimens were prospectively obtained with a pre-specified protocol and was annotated prior to knowledge of any correlative data. De Moel *et al* performed a study of 133 patients with melanoma with pre-ICI and post-ICI serum for autoantibody investigation^18^; however, clinical data were collected retrospectively. We had strong attribution of toxicity to the ICI (rather a random unrelated symptom) that was adjudicated by oncologists, and strengthened even further by using only the definite and probable toxicity events. The irAEs described in this clinical trial generally match those that have been previously described in the literature with regard to incidence, timing and distribution among organ systems.^32^ This study was designed with the assumption of about 30% seropositivity of ANA^33^ and/or RF and about 50% of patients experiencing a severe grade irAE,^5^ both of which were essentially met to test our primary hypothesis with adequate power. The different assay methodologies we used are complementary and provide data in different ways to answer similar questions. Lastly, we looked at whether there was any relation of autoantibodies to time to toxicity event, which has not been published in the literature yet.

There are limitations to our study, however, that should be addressed. Nine patients (15%) had missing 6-week plasma specimens, which limited the scope of testing for seroconversion and FCs. We decided to focus on nine major organ-specific irAE, but there are other irAE that occur, such as fatigue, anemia and acute kidney injury.^5 6^ These irAE were not of interest for this study either due to low incidence of occurrence or other possible attributable causes. For the microarray data, we did not have validated controls that translated signal intensity to results seen with commercial assays, thus, limiting the translation of this study to actual clinical practice. Because patients could experience multiple irAEs, there was no good control group to test true differential expression of autoantibodies among the various organ systems, and patient signal intensities could contribute to multiple organ groups. The majority (92%) of patients experienced an irAE of interest, which limited the size of the comparator group not experiencing an irAE. However, the five patients that did not experience any of the irAE of interest did have the least FC of autoantibodies from baseline to follow-up. It may be possible that other factors, such as the intrinsic malignancy or genetics, alter baseline autoantibody profiles, and thus play a role in irAE development. Given the small number of patients in our study and multiple comparisons, we may not have been powered to demonstrate an association between individual autoantibodies and organ-specific irAE, However, in almost every case of differentially expressed autoantibodies, the directionality of the association was a negative one. The microarray that was chosen contained antigens that are known to be associated with a variety of autoimmune diseases, but it was specifically enriched for autoantibodies found in SLE patients. While this may not be the most informative of arrays in the context of irAE, especially given the lack of SLE-like irAE observed after ICI,^34^ it does not rule out the possibility that there are other untested autoantibodies that will prove to have prognostic significance. Studies using a wider array of autoantigens with a larger number of patients with specific irAE will be needed to test this hypothesis. For commercial testing of ANA/RF/CCP, the study may have been underpowered to find true differences given the relatively low number of patients within organ-specific toxicity groups, especially for survival outcomes. We also did not analyze titers among the seropositive patients.

In conclusion, we found lower levels of discriminatory autoantibodies at baseline and a greater FC from baseline to 6 weeks in patients who experienced organ-specific irAE. This could suggest that the pretreatment balance between humoral and cellular immunity may impact irAE risk, and there may be a potential role for B cells in the development of irAE. However, how these data fully integrate into the T-cell centric immunopathology of irAE is yet to be determined. Comprehensive approaches to studying both humoral and cellular self-reactivity in irAE are clearly needed. Further studies should expand this type of analysis to confirm these hypotheses.

## Data Availability

All data relevant to the study are included in the article or uploaded as supplementary information.
