# Supplementary material for: Lower baseline autoantibody levels are associated with immune-related adverse events from immune checkpoint inhibition
Source: J Immunother Cancer. 2022 Jan 28;10(1):e004008. doi: 10.1136/jitc-2021-004008 (PMC8804686; doi:10.1136/jitc-2021-004008)
Supplement: Supplementary data [file jitc-2021-004008supp001.pdf]

Supplemental Tables/Figures

Table 1. Antigens included in the microarray autoantigen panel (UT Southwestern Core Lab)

Aggrecan	ssRNA	La/SSB	Ro/SSA(60KDa)
alpha Fodrin(Sptan1)	dsDNA	Laminin	S100
alpha-actinin	EBNA1	LC1	Scl-70
Amyloid	Elastin	LKM1	Sm
AQP4 recombinant	Entaktin EDTA	M2 antigen	Sm/RNP
BP1	Factor I	Matrigel	SmD
C1q	Factor P	MDA5	SmD1
Cardolipin	Factor B	Mi-2	SmD2
CENP-A	Factor D	Mitochondrial antigen	SmD3
CENP-B	Factor H	MPO	SP100
Chondroitin Sulfate C	Fibrinogen IV	Muscarinic receptor	Sphingomyelin
Chromatin	Fibrinogen S	Myelin basic protein (MBP)	SPR54
Collagen I	Fibronectin	Myelin-associated glycoprotein-FC	ssDNA
Collagen II	GBM (disso)	Myosin	T1F1 GAMMACollagen
Collagen III	Genomic DNA	Nucleolin	Thyroglobulin
Collagen IV	Gliadin (IgG)	Nucleosome antigen	TNFa
Collagen V	Glycated Albumin	Nup62	Topoisomerase I
Collagen VI	GP2	PCNA	TPO
complement C1q	gP210	Peroxiredoxin 1	TTG
complement C3	Histone H1	Phophatidylinositol	U1-snRNP-68
complement C3a	Histone H2A	PL-12	U1-snRNP-A
complement C3b	Histone H2B	PL-7	U1-snRNP-BB'
complement C4	Histone H3	PM/Scl-100	U1-snRNP-C
complement C5	Histone H4	PM/Scl-75	Vimentin
complement C6	Hemocyanin	POLB	Vitronectin
complement C7	Heparan HSPG	PR3	β2-glycoprotein I
complement C8	Heparin	Proteoglycan	β2-microglobulin
complement C9	Heperan Sulfate	Prothrombin protien	IgA - human and mouse
CPR antigen(human)	Histone (total)	Ribo phasphoprotein P1	IgE- human
Cytochrome C	Intrinsic Factor	Ribo phasphoprotein P2	IgG - human and mouse
Decorin-bovine	Jo-1	Ribo phasphoprotein P0	IgM - human and mouse
DGPS	KU (P70/P80)	Ro/SSA (52KDa)	Anti-IgG, IgA and anti-IgM

Table 2a. Patient characteristics

	ANA/RF/CCP negative at baseline (n=43)	ANA/RF/CCP positive at baseline (n=17)	*p-value*
Age, mean (SD)	59.6 (12.3)	62.4 (14.8)	0.71
Sex, male n (%)	18 (42)	4 (24)	0.24
Number of ICI cycles, median [IQR]	5 [3, 19]	6 [3, 10]	0.82
Any irAE experienced, n (%)	41 (95)	14 (82)	0.13
Time to irAE (weeks), median [IQR]	3.7 [1.4, 6.4]	2.8 [1.5, 9]	0.56
Time to irAE, <6 weeks, n (%)	13 (30)	4 (24)	1.00
Severe irAE (grade 3-5), n (%)	18 (42)	10 (59)	0.26
Total number of irAE, median [IQR]	3 [2, 5]	3 [2, 4]	0.95

Table 2b. Organ-specific irAE

	ANA/RF/CCP negative at baseline (n=43)	ANA/RF/CCP positive at baseline (n=17)	*p-value*
Rash/pruritis, n (%)	19 (44)	4 (24)	0.16
Diarrhea/colitis, n (%)	12 (28)	9 (53)	0.08
Myocarditis, n (%)	2 (5)	1 (6)	1.00
Myalgia/myositis, n (%)	5 (12)	1 (6)	0.66
Hepatitis, n (%)	16 (37)	6 (35)	1.00
Thyroid, n (%)	14 (33)	0 (0)	**0.006**
Arthritis, n (%)	4 (9)	2 (12)	1.00
Sicca, n (%)	7 (16)	1 (6)	0.42
Non-thyroid endocrine, n (%)	14 (33)	3 (18)	0.35

Table 2. (A) Differences in patient characteristics between those positive and negative for ANA/RF/CCP at baseline. (B) Differences in organ-specific irAE events between those positive and negative for ANA/RF/CCP at baseline

Figure 1 A-I

A

Hepatitis

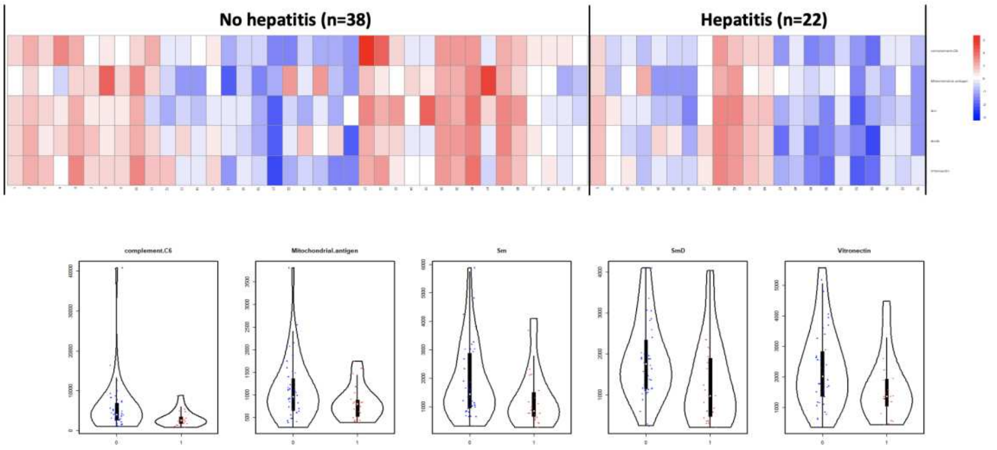

Diarrhea/Colitis

B

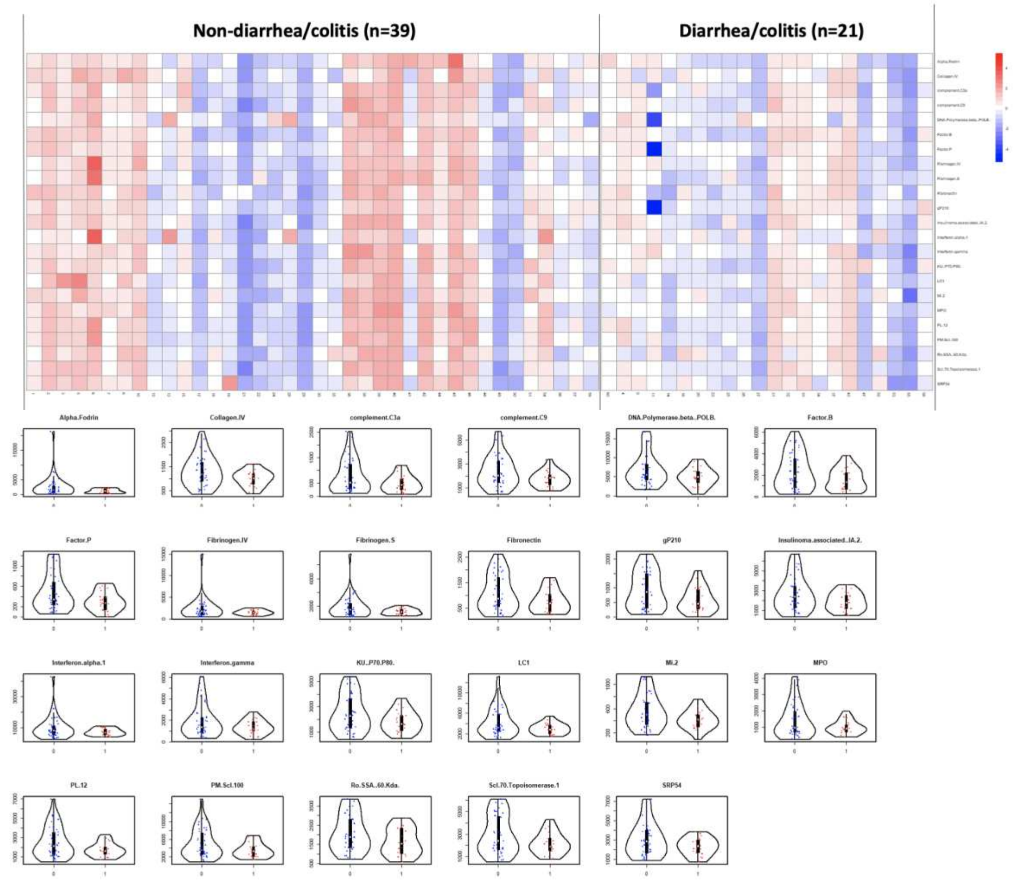

C

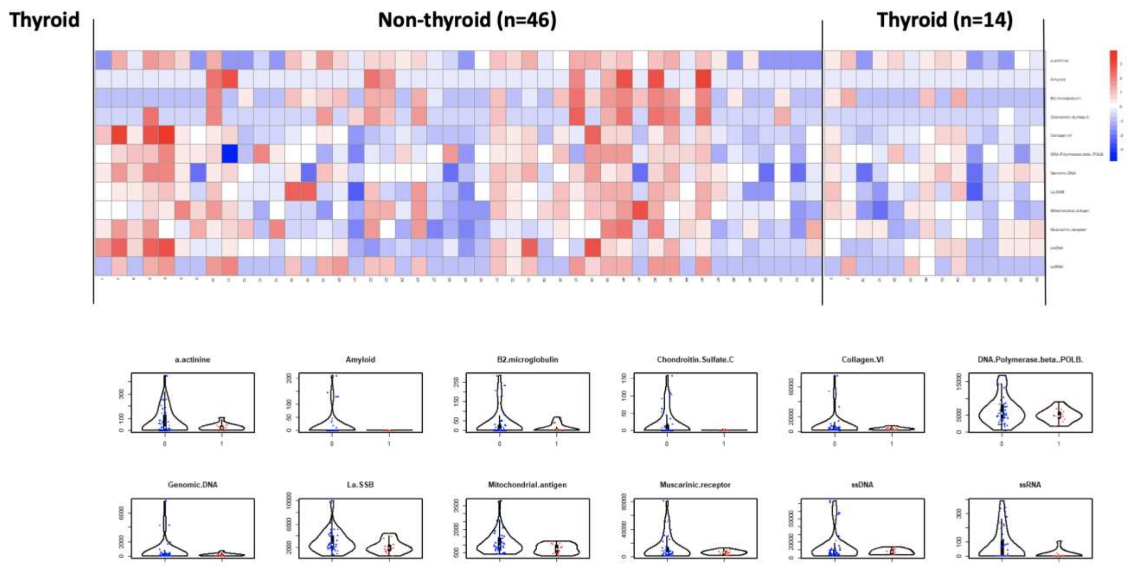
Thyroid
Non-thyroid (n=46)
Thyroid (n=14)
a.actinine
Amyloid
B2.microglobulin
Chondroitin.Sulfate.C
Collagen.VI
DNA.Polymerase.beta..POLB.
Genomic.DNA
La.SSB
Mitochondrial.antigen
Muscarinic.receptor
ssDNA
ssRNA

D

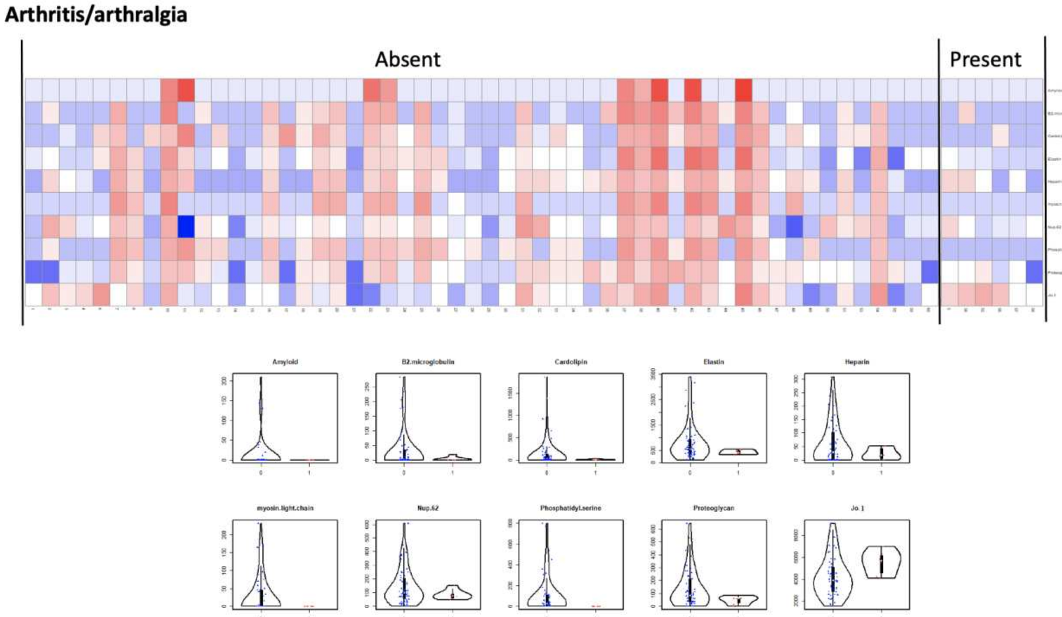
Arthritis/arthralgia
Absent
Present
Amyloid
B2.microglobulin
Cardiolipin
Elastin
Heparin
myosin.light.chain
Nup.62
Phosphatidyl.serine
Proteoglycan
Jo.1

E

Myocarditis (n=3)

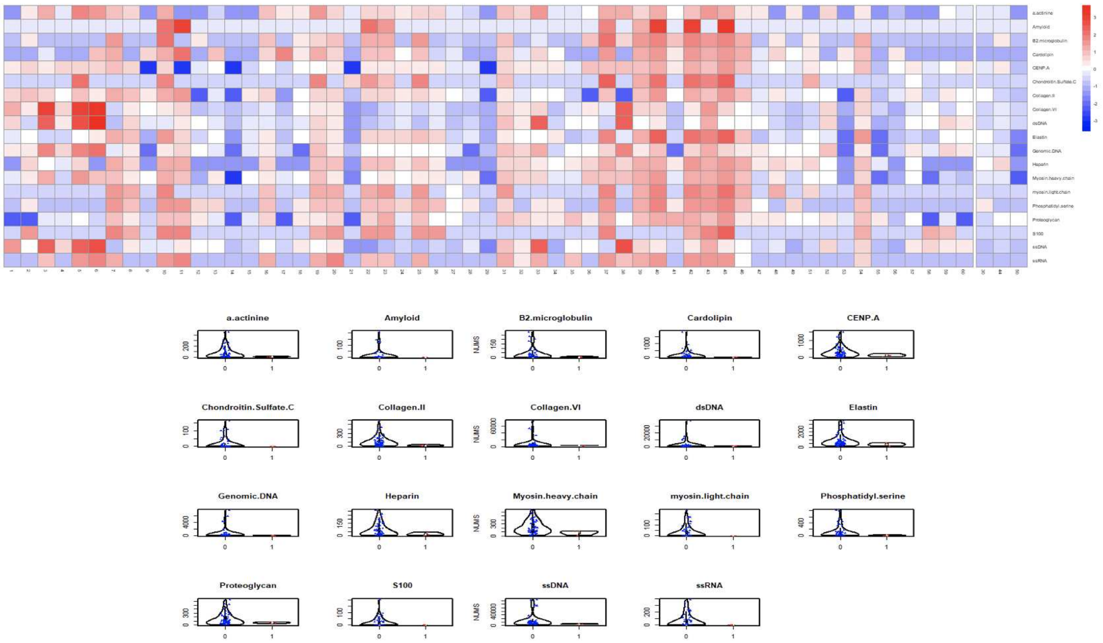

F

Myalgia

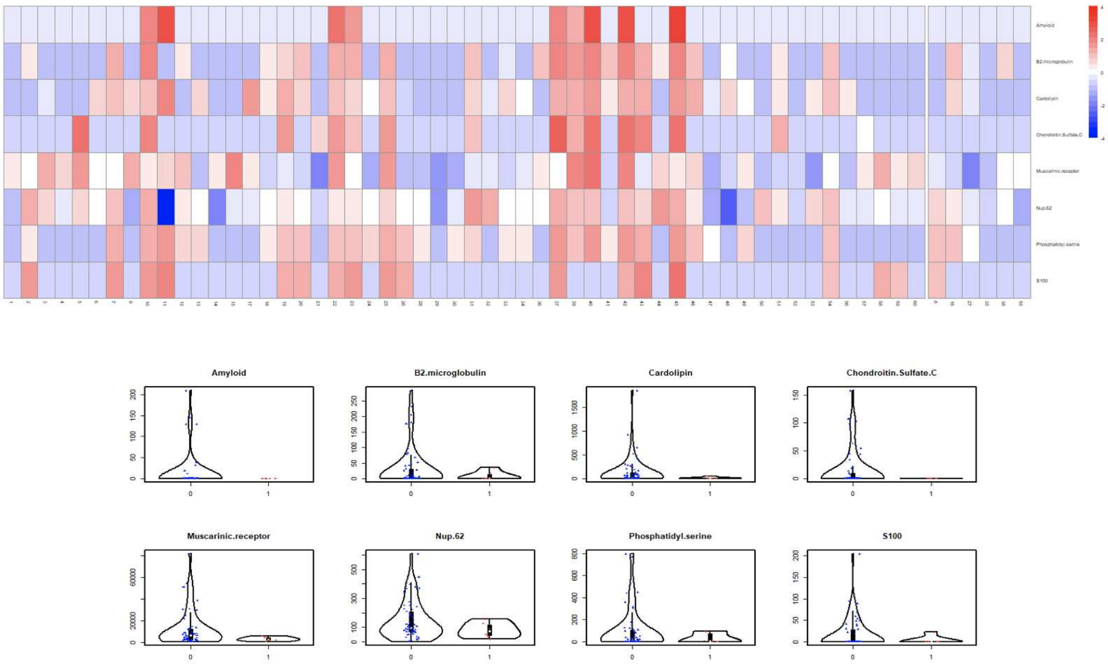

G

Sicca

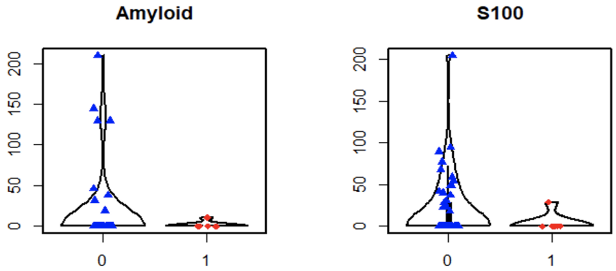

Non-thyroid endocrine

H

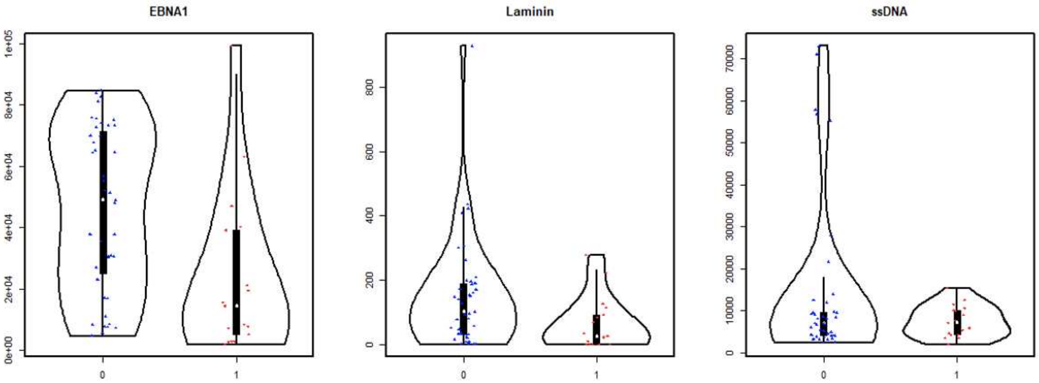

Timing (<6 weeks =0)

I

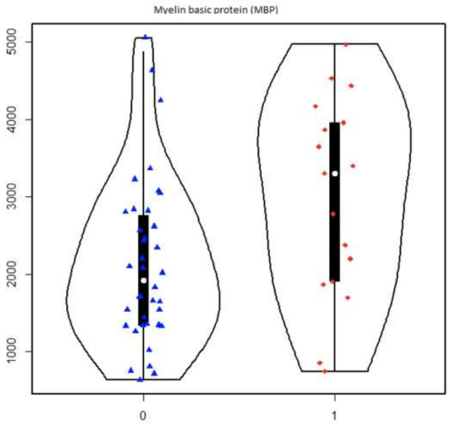

Figure 1A-I. Violin plots identifying antigens with differential baseline IgG expression among patients experiencing each organ-specific irAE (A-H), as well as timing of early vs. late (I).

BMJ Publishing Group Limited (BMJ) disclaims all liability and responsibility arising from any reliance placed on this supplemental material which has been supplied by the author(s)

Figure 2

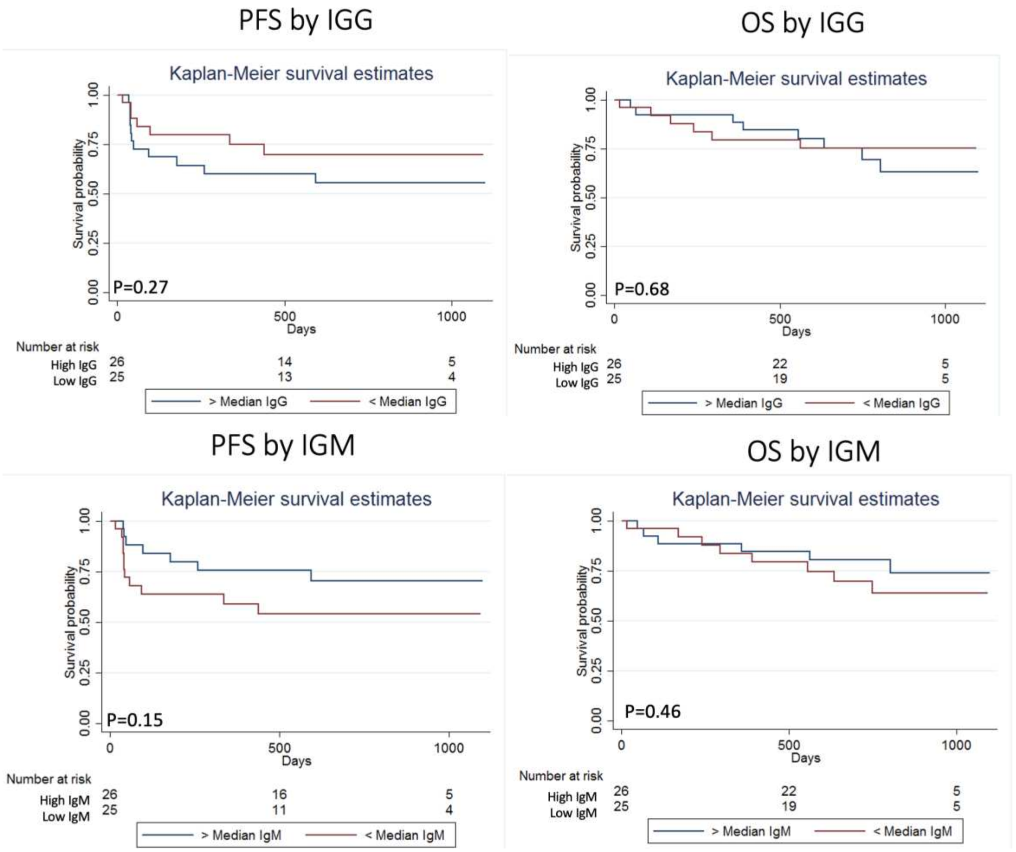

Figure 2. Progression free survival (PFS) and overall survival (OS) curves comparing top half (>median) and bottom half (<median) of total IgG and IgM concentration in plasma at baseline.
